# Dual Crosslinking of Alginate Outer Layer Increases Stability of Encapsulation System

**DOI:** 10.3389/fchem.2020.575278

**Published:** 2020-11-12

**Authors:** Sami I. Somo, Jacob M. Brown, Eric M. Brey

**Affiliations:** ^1^Biomedical Engineering Department, Illinois Institute of Technology, Chicago, IL, United States; ^2^Biomedical Engineering Department, University of Texas at San Antonio, San Antonio, TX, United States

**Keywords:** alginate (PubChem CID: 91666324), islets, crosslinking, type 1 diabetes (or diabetes), encapsulation

## Abstract

The current standard treatment for Type 1 diabetes is the administration of exogenous insulin to manage blood glucose levels. Cellular therapies are in development to address this dependency and allow patients to produce their own insulin. Studies have shown that viable, functional allogenic islets can be encapsulated inside alginate-based materials as a potential treatment for Type 1 diabetes. The capability of these grafts is limited by several factors, among which is the stability and longevity of the encapsulating material *in vivo*. Previous studies have shown that multilayer Alginate-Poly-L-Ornithine-Alginate (A-PLO-A) microbeads are effective in maintaining cellular function *in vivo*. This study expands upon the existing encapsulation material by investigating whether covalent crosslinking of the outer alginate layer increases stability. The alginate comprising the outer layer was methacrylated, allowing it to be covalently crosslinked. Microbeads with a crosslinked outer layer exhibited a consistent outer layer thickness and increased stability when exposed to chelating agents *in vitro*. The outer layer was maintained *in vivo* even in the presence of a robust inflammatory response. The results demonstrate a technique for generating A-PLO-A with a covalently crosslinked outer layer.

## Introduction

Diabetes mellitus is a metabolic disease characterized by defective insulin secretion by pancreatic β-cells in response to glucose stimulation. The current pharmacological treatment of Type I diabetes involves administration of exogenous insulin in response to blood glucose levels. Glucose levels are either self-monitored or tested by an implanted device. While implanted systems have dramatically improved in recent years, a level of control equivalent to endogenous insulin secretion from β-cells cannot be achieved (Scharp and Marchetti, 2014; Kollmer et al., 2016; Dinnyes et al., 2020). Over time, this can lead to complications such as kidney failure, loss of vision, foot ulcers, and limb amputation (Litwak et al., 2013).

Cell-based therapies have been proposed as an alternative to exogenous insulin therapy. Endocrine β-cell clusters, islets, within the pancreas have been implanted into patients to restore normal pancreatic function. Despite promising results achieved with naked islets, the lack of available donor tissue, the number of islets needed per patient, the need for immunosuppressants, and eventual loss of β-cell function over time has hampered islet transplantation. Investigation of biomaterials for the encapsulation and immunoisolation of islets is a strategy to protect these cells from the host autoimmune system and increase their longevity.

Alginate has been investigated extensively as an encapsulation medium for β-cell islets. These microencapsulation systems have been shown to improve the survival and function of islet grafts implanted in both genetically and chemically induced diabetic animal models (Omer et al., 2003; Qi et al., 2008; Rengifo et al., 2014; Lawandi et al., 2015; Vegas et al., 2016). In clinical trials, these systems have enabled some patients to reduce their exogeneous insulin usage for extended periods of time. The efficacy and duration of the improvement varied, however, with some patients requiring multiple transplants and none becoming completely insulin independent. The cause of the inconsistency in patient outcomes is hard to discern, as it might arise from the use of varying alginate compositions, islet sources, implantation sites, and/or patient to patient variation (Calafiore et al., 2005; Kollmer et al., 2016). While the primary reason for graft failure is unknown, one identified mechanism is the breakdown of the alginate microbeads following implantation. Alginate microbeads which are only ionically crosslinked can become unstable due to calcium chelation and sodium ion exchange (van Raamsdonk et al., 2002; Strand et al., 2017). Breakdown of ionically crosslinked beads has been observed in *in vivo* models (Ibarra et al., 2016).

**Figure 1:**
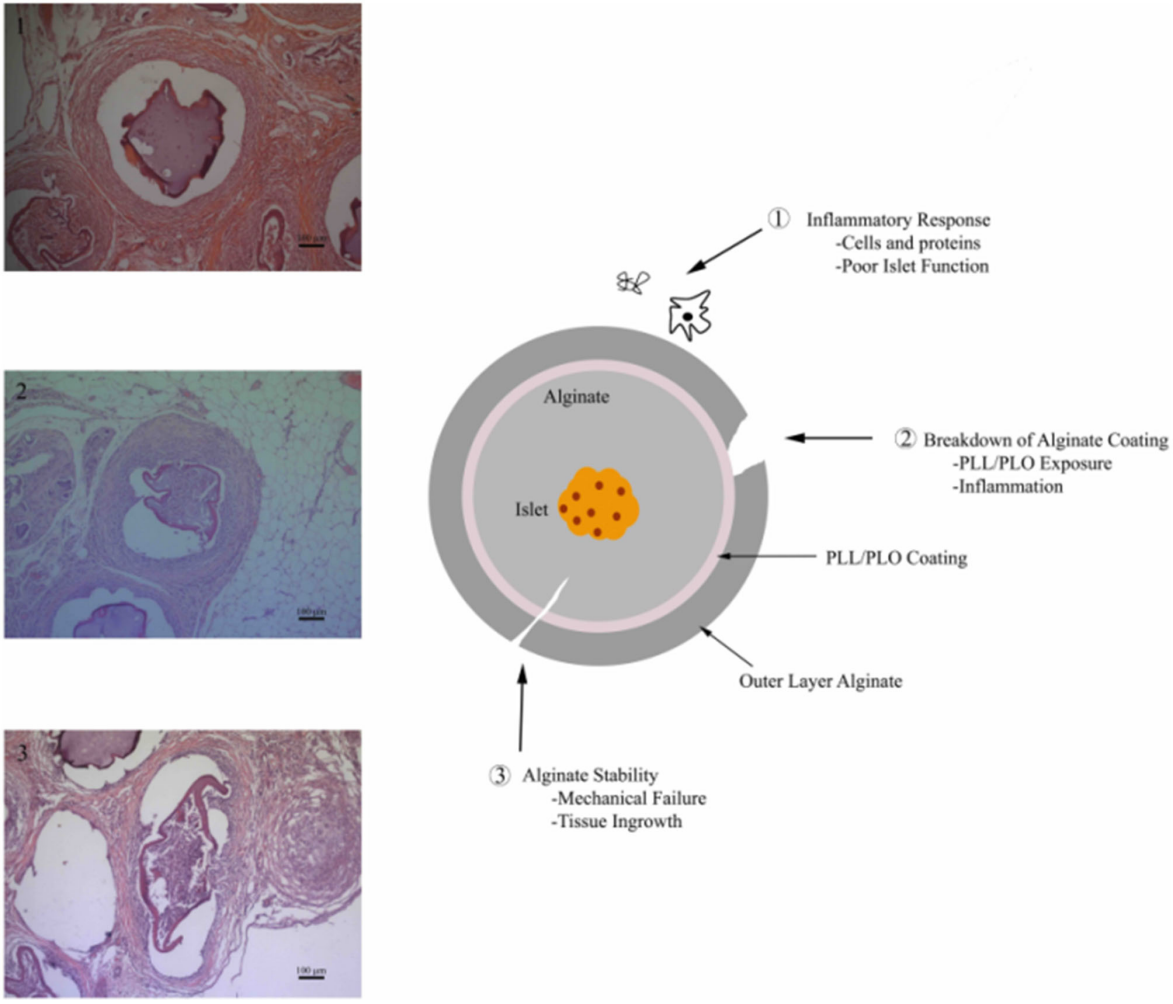
Alginate microbead failure mechanisms (1) robust inflammatory response leading to encapsulation of the material, (2) failure or breakdown of alginate coating exposing the underlying positively charged permselective membrane resulting in a chronic inflammatory response, and (3) alginate breakdown via chemical degradation or mechanical failure.

Alginate is relatively stable biomaterial, but breakdown can occur due to a number of factors, including inflammation or mechanical stress (Figure 1). Implanting alginate microbeads in the body triggers the foreign body response, which ultimately leads to the growth and differentiation of immune cells and fibroblasts around the microbeads. This fibrous capsule has the potential to not only disrupt the transport of nutrients, compromising the function and survival of the islets, but can also directly lead to the degradation of the microbeads (Anderson et al., 2008; Veiseh et al., 2015). The collection of inflammatory cells on the surface of the bead may also lead to the degradation of the alginate crosslinks by increasing reactive oxygen species production (de Vos et al., 2003a). These effects may be modulated by adjusting the size, shape, and polycation layer concentration of the microbeads, among other attributes (Ponce et al., 2006; Veiseh et al., 2015). The implant can also be subjected to different mechanical stresses depending on where it is located. Beads implanted within the peritoneal cavity experience different mechanical stresses compared to those implanted within the omentum (Dufrane et al., 2006). Increasing stability of the microbeads and their resistance to the inflammatory response could increase their effective duration when applied as a therapy for diabetes.

Alginate microbeads currently used for β-cell encapsulation typically consist of three separate individual layers. The first is an inner layer alginate core containing β-cell islets (Somo et al., 2017). The second layer is a semipermeable monolayer which allows oxygen and nutrients to diffuse in and out, while preventing the encapsulated cells from being exposed to larger molecules or other cells. Finally, this permselective layer is covered with a second alginate layer (ranging from a single monolayer to ~100 μm thickness) to prevent cell interactions with the polycationic semipermeable membrane, which can lead to increased inflammation. The outer layer can also be used to deliver molecules that locally modulate the inflammatory response angiogenesis (Khanna et al., 2010, 2013). Dual layer alginate microbeads with an intermediate Poly-L-ornithine microbeads, referred to here as A-PLO-A (Alginate-Poly-L-Ornithine-Alginate), can protect islets and enable long-term function (Kollmer et al., 2016). However, any breakdown of the outer alginate layer may lead to failure of the implants.

Several investigators have introduced covalent crosslinks into alginate-based materials to increase stability (Hall et al., 2011; Breger et al., 2015; Hillberg et al., 2015). For example, methacrylated single-layer alginate microbeads were shown to enhance stability of microspheres *in vitro* and *in vivo* when used to dual crosslink alginate microbeads, without affecting cell viability (Somo et al., 2018). However, A-PLO-A microbeads were not used in these studies. In addition, the alginate used for the studies was a crude alginate solution which is poorly characterized and has limitations regarding its interaction with the polycationic membrane (Thu et al., 1996; Bhujbal et al., 2014). In addition, previous studies have not focused on stabilizing the outer layer, which is the interface between the biomaterial and the host, playing a significant role in modulating the inflammatory response.

In this study, the dual (simultaneously ionically and covalently) crosslinking of the outer alginate layer was investigated as a method for increasing the stability of multilayer A-PLO-A microcapsules. The *in vivo* portion of the study was performed without encapsulating cells to reduce the risk of complications, as the primary intent was to compare differing outer layer compositions, which would not contain cells in the final application. The effect of the photopolymerization process on MIN6 cells, routinely encapsulated using these systems, was assessed as part of a previous study. Their viability was maintained above 90% at methacrylation efficiencies of up to 4% (Somo et al., 2018). Methacrylated low viscosity ultra-pure sodium alginate with high guluronic acid content (LVG) was used as the alginate base for the outer layer. Methacrylation allowed for the covalent crosslinking of the LVG alginate, in addition to the normally ionically crosslinked mechanism. A-PLO-A microbeads with a methacrylated LVG outer layer exhibited controlled outer layer thickness (~170 μm) and increased stability when exposed to CaCl_2_ chelating agents. The outer layer was maintained *in vivo* even in the presence of a robust inflammatory response. These results demonstrate that A-PLO-A microbeads with a covalently crosslinked outer layer exhibit increased stability.

## Materials and Methods

### Materials

Low viscosity (20–200 mPaS) ultra-pure sodium alginate with high mannuronic (LVM) and high guluronic acid (LVG) contents were purchased from Nova-Matrix (75–200 kDa, G/M ratios of 1 and 1.5, respectively). 2-hydroxy-2-methylpropiophenone (Irgacure 1173), poly-l-ornithine (PLO) hydrochloride (MW: 15,000–30,000), 2-morpholinoethanesulfonice acid (MES), N-Hydroxysuccinimide (NHS), 1-ethyl-3-(3-dimethylaminopropyl)-carbodiimide hydrochloride (EDC), lipopolysaccharide (LPS) from Escherichia coli 0111:B4, Dulbecco's Modified Eagle's medium (DMEM), Dulbecco's phosphate-buffered saline (DPBS), and 2-mercaptoethanol were purchased from Sigma-Aldrich (St. Louis, MO). Fetal Bovine Serum (FBS) and penicillin-streptomycin was purchased from Life Technologies (Waltham, MA). MIN6 cell line was purchased from AddexBio (San Diego, CA). 2-Aminoethyl methacrylate hydrochloride (AEMA) was purchased from Polysciences (Warminster, PA). Live/Dead kit was purchased from Invitrogen (Eugene, OR). Solutions for alginate microbead fabrications were made using the following chemicals: HEPES, NaCl, MgCl2 (Fisher Scientific); CaCl2 (Acros).

### Synthesis and Characterization of LVG Methacrylated Alginate

Methacrylated LVG alginate was synthesized based on the modification of a previous protocol (Somo et al., 2018). Briefly, 1% w/v LVG alginate was dissolved in a buffer consisting of 0.5 M NaCl and 50 mM MES. NHS and EDC were added to the mixture sequentially and mixed for 5 min. Ninety five milligram of AEMA was added to the mixture and the reaction maintained at room temperature for 24 h. AEMA was added to the mixture of EDC and NHS in a concentration that maintained a molar ratio of NHS:EDC:AEMA equal to 1:2:1. After 24 h, the reaction was precipitated with excess acetone using a Buchner funnel through 5 μm filter paper. The product was recovered and dissolved in 50 mL of deionized (DI) water and precipitated again with acetone. The product was dissolved in 50 mL DI water and dialyzed (MWCO 3500) against DI water for 3 days. The methacrylated alginate solution was filtered with a 0.22 μm filter and lyophilized. As a control, unmodified alginate (LVG) was processed in the same manner in the absence of AEMA. ^1^H nuclear magnetic resonance (NMR) was performed to evaluate methacrylation. Methacrylated alginate (15 mg) was dissolved in 1 mL of deuterium oxide and placed in NMR tubes. The NMR spectrum of the methacrylated alginate was recorded on a Bruker 300 Ultrashield NMR spectrometer. The methacrylation efficiency (ME) was determined as the ratio of the integrals for the methylene protons of methacrylate (δ5.3–δ5.8 ppm) to alginate protons (δ3.5-δ4.0 ppm) (Figure 3A).

### Fabrication of Multilayered Alginate Microbeads

**Figure 2:**
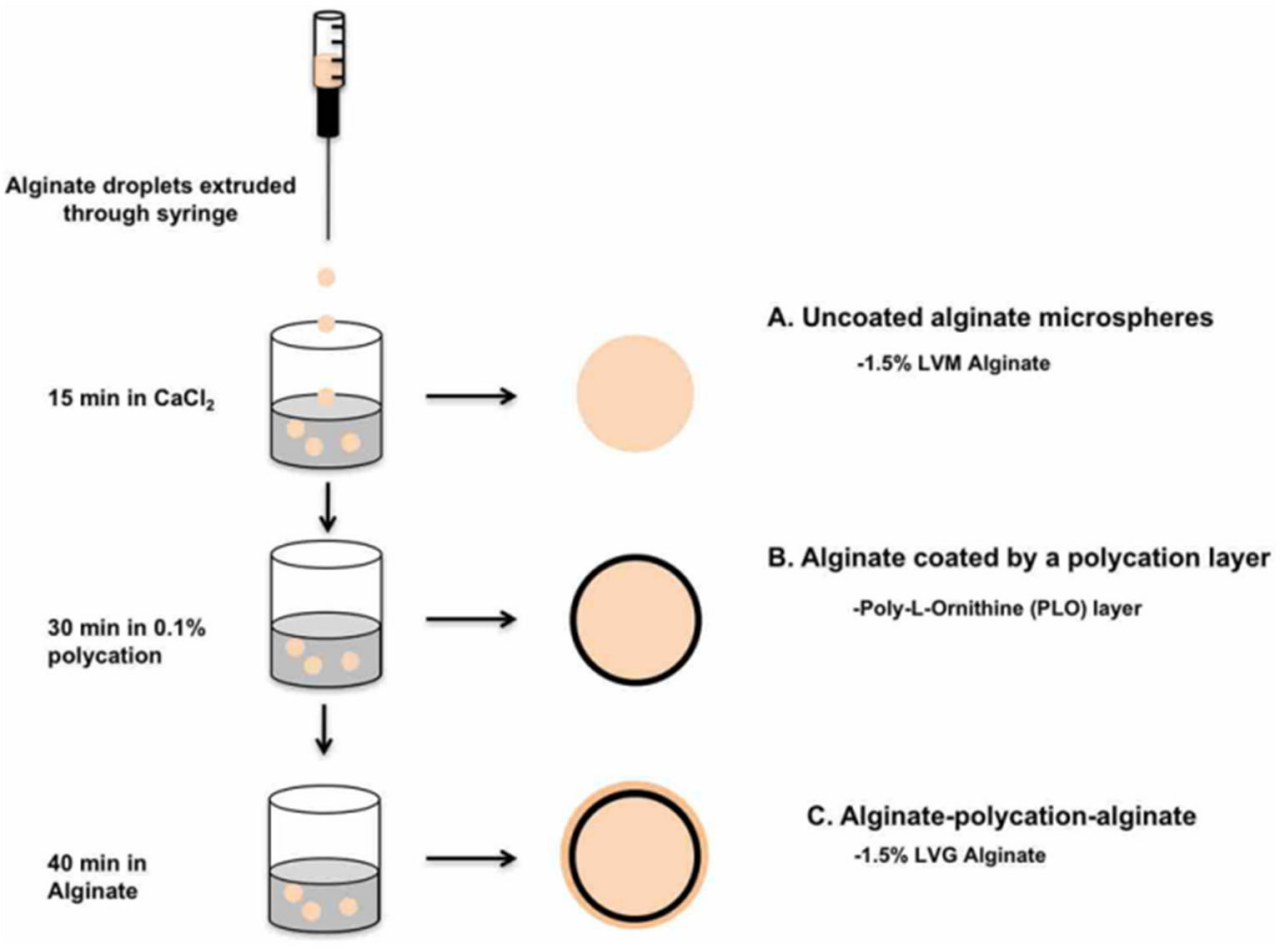
Procedure for fabrication of A-PLO-A microbeads. **(A)** Uncoated alginate microspheres formed in CaCl_2_ bath followed by **(B)** incubation in 0.1% polycation solution. **(C)** Outer later formed by incubation of 1.5% w/v LVG or 1.5% w/v methacrylated LVG for 40 min.

Microbeads were prepared under sterile conditions using a standard method of injection into a cationic crosslinking solution. LVM alginate (1.5% w/v) was dissolved in a solution consisting of 25 mM HEPES, 118 mM NaCl, 5.6 mM KCl, 2.5 mM MgCl_2_. The dissolved precursor was first sterilized by extrusion through a 0.22 μm syringe filter. The filtered precursor was then extruded through a 1 mL syringe with a blunt 20-gauge needle into 15 mL of a crosslinking solution consisting of 100 mM CaCl_2_ and 10 mM HEPES. The beads were incubated in the crosslinking solution for 15 min. After 15 min, the beads were washed three times with 2 mM CaCl_2_ and saline (0.9% NaCl) for 2 min. The microbeads were transferred into a 0.1% (w/v) solution of PLO in normal saline and rocked for 30 min, resulting in the formation of a PLO coating. Three washes were performed with 2 mM CaCl_2_ and 0.9% NaCl solution to remove residual PLO. The PLO-coated microbeads were dried and blotted with a kimwipe to remove excess water, then transferred into an alginate solution. At this point, beads were divided into two groups, one with a regular LVG alginate outer layer, and the other with a methacrylated LVG outer layer. The microbeads were incubated in the alginate solution for 40 min, allowing the alginate time to interact with the PLO layer. Excess alginate solution was removed, and the outer layer was crosslinked in a solution of 22 mM CaCl_2_ for both groups. The crosslinking solution for the methacrylated LVG beads additionally contained 0.05% (w/v) Irgacure 1173 (photoinitiator), and the beads were exposed to UV light for 5 min for photocrosslinking. Two additional washes were performed with 2 mM CaCl_2_ and 0.9% NaCl solution to remove unbound alginate. After synthesis, the microbeads were imaged using Axiovert 200x inverted microscope (Carl Zeiss MicroImaging) with a 5x objective, and the size of the outer layer quantified with AxioVision. Ten microbeads were measured per condition, and the experiments were repeated three times. A schematic of the bead synthesis steps is shown in Figure 2.

**Figure 3:**
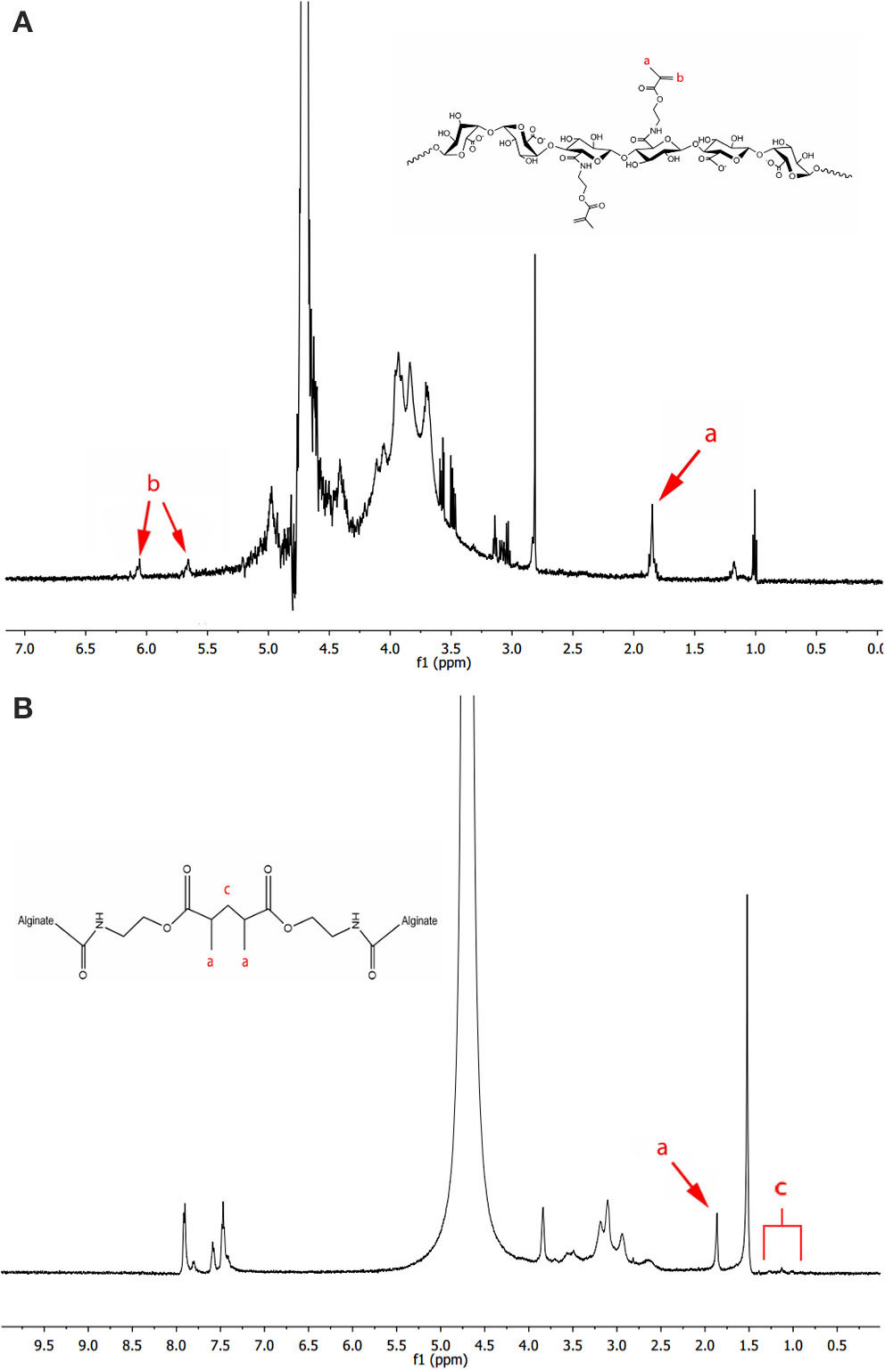
^1^H NMR spectra of **(A)** methacrylated LVG with the existence of methyl (a) and methylene (b) protons of the methacrylate, and **(B)** photocrosslinked methacrylated LVG in deuterium oxide. The methylene peaks (b) disappear after alginate has been photocrosslinked.

The NMR spectrum of crosslinked methacrylated alginate was obtained by manually separating the outer layer from dual layer dual crosslinked beads. Once separated, the combined outer layers were placed into an NMR tube, lyophilized, and finally rehydrated with D_2_O (Figure 3B).

Scanning electron microscopy (SEM) was used to evaluate the surfaces of the microbeads. Alginate microbeads were incubated in 2.5% glutaraldehyde at 4°C for 2 h. The microbeads were then washed 3 times with distilled water. Microtubes containing the microbeads and distilled water were frozen in liquid nitrogen and then lyophilized overnight. Randomly selected microbeads were deposited on carbon conductive tape adhered to SEM aluminum stubs. The beads were analyzed using a Phenom PRO Desktop SEM (Phenom-World, Netherlands) operated at 10 kV.

### Stability Assessment of Outer Alginate Layer

To assess the stability of alginate beads *in vitro*, microbeads were incubated in a solution consisting of 55 mM sodium citrate, 50 mM NaCl, and 30 mM EDTA. Alginate microbeads prepared as described above were incubated for 24 h at 37°C in a humidified atmosphere. Microbeads were imaged before and after the addition of the outer layer for assessment using an Axiovert 200x inverted microscope (Carl Zeiss MicroImaging) with a 5x objective. The size of the outer layer was quantified with AxioVision. To determine long term swelling properties, fresh alginate beads were incubated in saline solution at 37°C in a humidified atmosphere over a period of 40 days. The media was replaced after the beads were imaged. Four points along the edge of the inner layer were selected to generate a circle approximating the shape of the inner layer, and then the outer layer was determined in the same way. The computer-generated diameter of the outer layer was subtracted from the inner layer to determine the thickness of the outer layer (*n* = 15 for each group).

### *In vivo* Stability Model

Animal experiments were carried out using procedures approved by Illinois Institute of Technology's Institutional Animal Care and Use Committee. An omentum pouch model was used to evaluate microbeads *in vivo*. Two alginate conditions were examined: (1) A-PLO-A microbeads prepared under sterile conditions with an ionic-crosslinked outer layer and (2) dual crosslinked outer layer alginate microspheres. The outer layer was 1.5% (w/v) concentration for both. A total of 16 animals were used, 4 per group per time point (1 and 3 weeks). Male Sprague Dawley rats (300–400 g, *n* = 4; Envigo) were anesthetized initially with 5% isoflurane. Body temperature was maintained at 37°C with a heating pad, and anesthesia was maintained with a 2% isoflurane/oxygen gas mixture during the procedure. Each animal had their abdomen shaved, and skin scrubbed with isopropyl alcohol, followed by a povidone-iodine antiseptic solution. The omentum was surgically exposed by midline laparotomy. First, the skin was separated from muscle and a ~2-inch incision was made. Next, the underlying muscle was cut to expose the organs and the greater omentum was carefully pulled from the abdomen. Using 4–0 Ethilon suture, a purse-string suture was positioned around the edges of the omentum to create a pouch for the beads. Fifty alginate beads were placed on the anterior surface of the exposed omentum. LPS (100 μl of 50 μg LPS dissolved in 1 mL saline) was directly injected onto the anterior surface of the omentum to stimulate an inflammatory challenge (Somo et al., 2018). Afterwards, the pouch was folded over and sutured to secure the beads inside. The underlying muscle and then skin were closed with 4–0 Ethilon suture. After surgery, the animals were allowed to recover, and were monitored closely. At each time point (week 1 and week 3) the omenta were explanted, fixed in formalin, and prepared for histological characterization and imaging. The tissues were processed for histology using standard methods and tissues were paraffin embedded. Samples were sectioned at 5 μmm thickness and stained with hematoxylin and eosin (H&E) and Masson's Trichrome. Tissue sections were imaged using an Axiovert 200 inverted microscope (Carl Zeiss MicroImaging). The outer layers of the beads were observed and measured using the AxioVision software (Carl Zeiss MicroImaging), for each of five selected beads from each condition, five distances were quantified between the inner and outer layers.

### Statistical Analysis

Statistical analysis was performed using GraphPad Prism. All data are expressed as mean ± standard deviation. *In vitro* data were analyzed using one-way ANOVA with a Tukey's post-test for normally distributed data. Values of *p* < 0.05 were considered statistically significant.

## Results

### Methacrylation Efficiency

Methacrylated alginate was produced by reacting LVG alginate with AEMA using NHS/EDC chemistry. The ^1^H NMR spectra of methacrylated alginate is shown in Figure 3A. Peaks associated with AEMA can clearly be identified in the spectra. The methacrylated alginate had peaks corresponding to methylene (δ5.7 and δ6.1) and methyl (δ1.9) groups that are formed by the reaction with AEMA. The procedure resulted in a methacrylation efficiency of 2.16% ± 0.33. The ^1^H NMR of methacrylated LVG alginate after covalent crosslinking is shown in Figure 3B. After exposure to UV in the presence of an appropriate initiator, the absence of the methylene peaks in the spectrum indicates the reaction of the AEMA groups.

### Microbead Fabrication and Size

**Figure 4:**
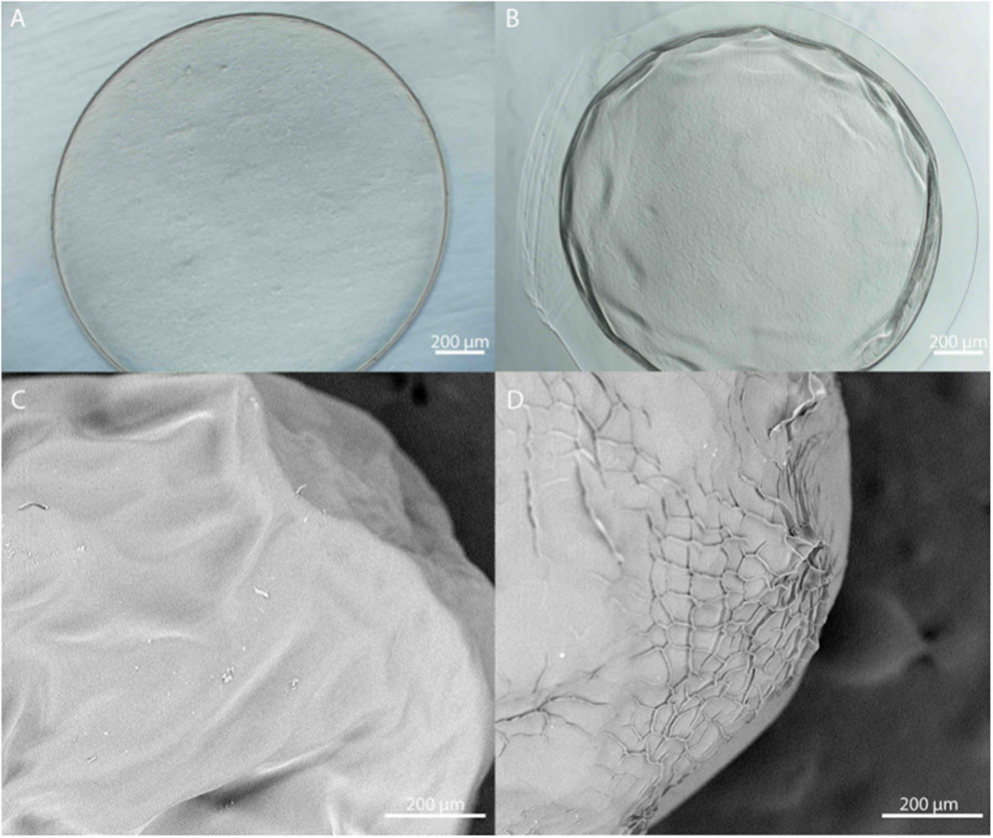
**(A,B)** Phase contrast and **(C,D)** SEM images of PLO coated alginate microbeads **(A,C)** prior to and **(B,D)** after formation of the outer layer.

**Figure 5:**
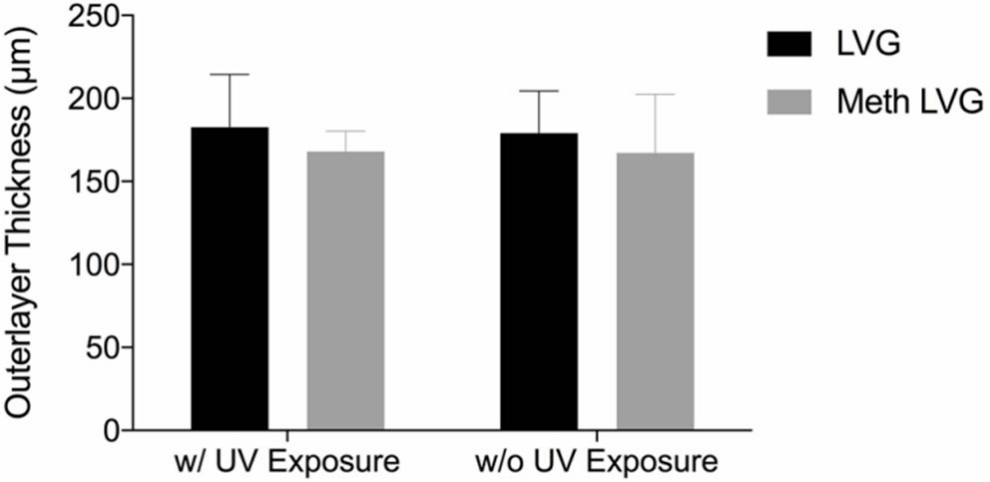
The size of the outer layer of multilayered alginate microbeads was not statistically different with LVG and methacrylated LVG with and without UV exposure.

Microbeads were synthesized using 1.5% (w/v) LVM alginate for the inner core (Figure 1) and two formulations for the outer core: LVG and methacrylated LVG. A distinct alginate layer is present on the outside of the beads after incubation in the polycation bath (Figure 4A). Following incubation in LVG or methacrylated LVG alginate, the outer layer could be observed surrounding the alginate core and PLO layer (Figure 4B). The outer layer exhibited a roughness (Figure 4D) that was not observed in the PLO surface alone (Figure 4C) The size of the alginate outer layer did not vary significantly between the two alginate compositions or with UV exposure (Figure 5). The outer layer sizes were 179 ± 25 μm, 182 ± 31 μm, 167 ± 35 μm and, 168 ± 12 μm for LVG, LVG exposed to UV, methacrylated LVG, and methacrylated LVG exposed to UV, respectively (*n* = 10).

### Stability and Long-Term Swelling

**Figure 6:**
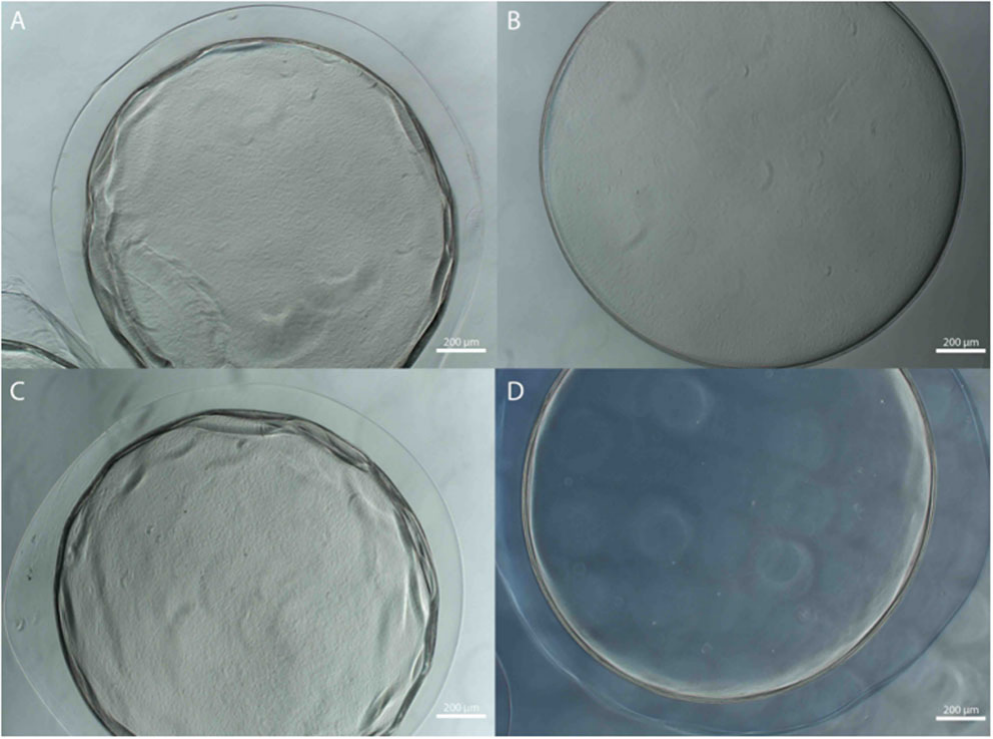
Multilayered alginate microbeads with **(A,B)** LVG and **(C,D)** methacrylated LVG outer layer **(A,C)** before and **(B,D)** after exposure to sodium citrate.

**Figure 7:**
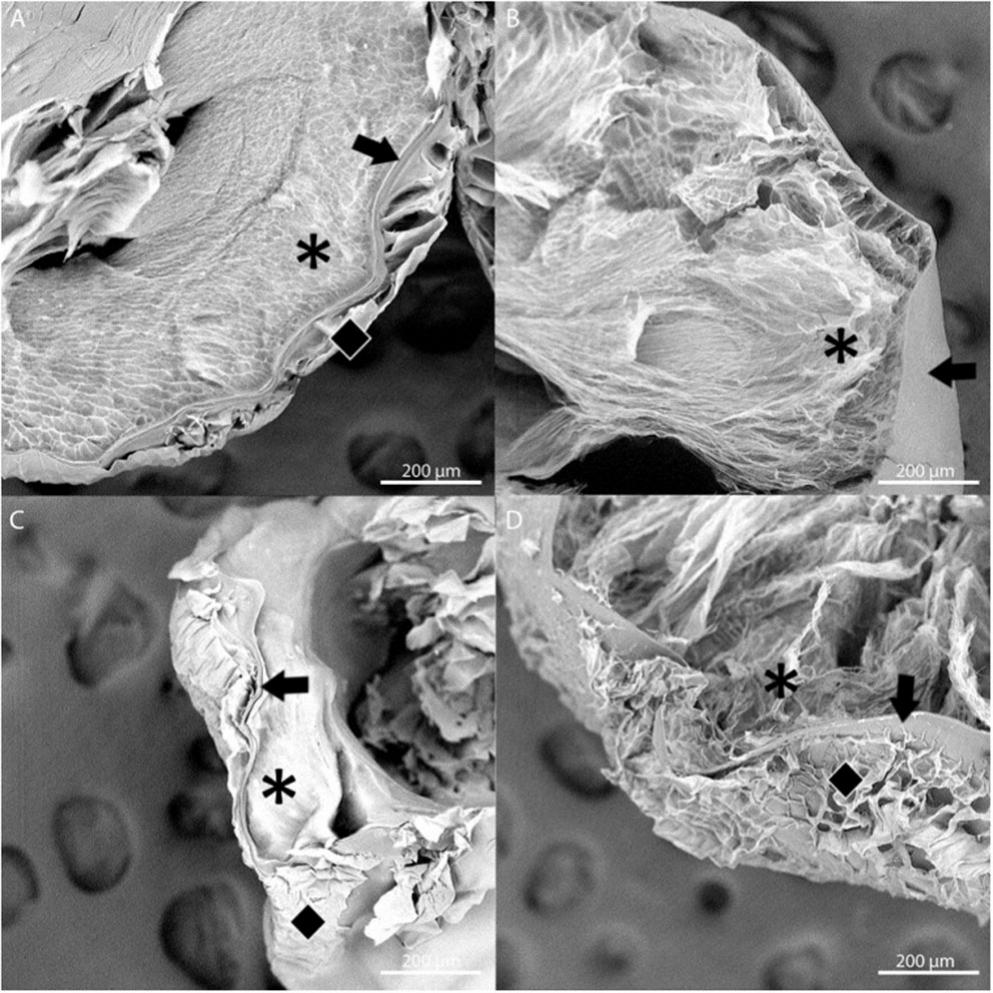
SEM images of multilayered alginate microbeads with LVG outer layer **(A,B)** and methacrylated LVG outer layer **(C,D)** before **(A,C)** and after **(B,D)** exposure to sodium citrate for 4 hours. Layers are represented by symbols: inner core (asterisk), PLO layer (arrow), outer layer (diamond). Three distinct layers are observed with methacrylated LVG outer layer **(D)** after exposure to sodium citrate.

The stability of the alginate outer layer was first assessed *in vitro* based on incubation in sodium citrate, a chelating agent. A-PLO-A microbeads were prepared with an LVG outer layer or a dual crosslinked methacrylated LVG outer layer and samples placed in a solution of 55 mM sodium citrate, 50 mM NaCl, and 30 mM EDTA at 37°C room temperature for 4 h (Figure 6). The outer layer of the standard outer layer conditions (LVG) was absent after sodium citrate exposure (Figure 6A). Outer layers formed by crosslinked methacrylated LVG microbeads exhibited greater integrity without any evidence of breakdown (Figure 6D). SEM cross-sectional images of the beads are presented in Figure 7. Upon dissolution of outer layer, the smooth PLO surface is exposed, and only two distinct layers are seen, the inner alginate core and the smooth PLO layer (Figure 7B). However, the images of methacrylated LVG retained the distinct outer layer after sodium citrate exposure (Figure 7D).

**Figure 8:**
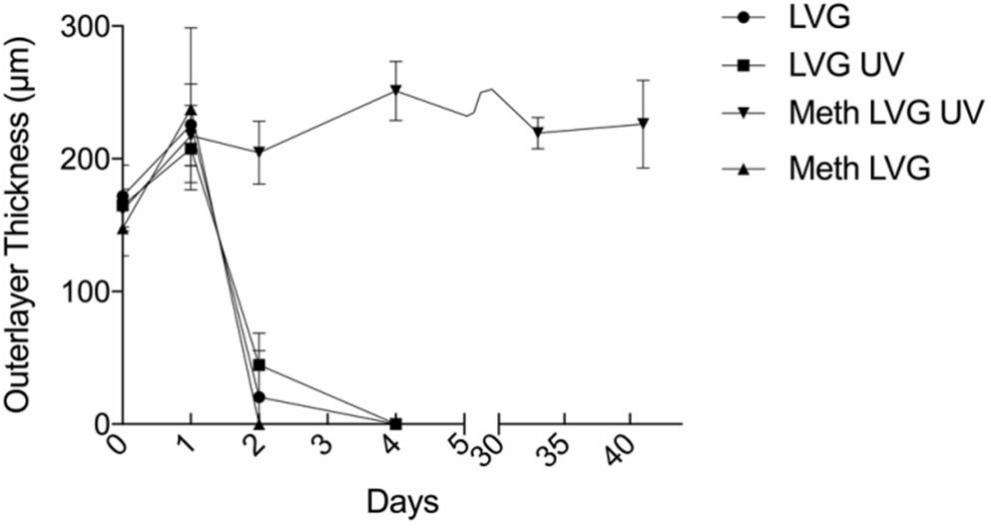
The size of the outer layer of the microbeads vs. time during incubation in a solution of 0.9% NaCl solution at 37° C. The methacrylated LVG exposed to UV swelled initially and remained stable for over a period of 40 days.

The time-dependent behavior of the microbeads was evaluated by incubation in a 0.9% NaCl solution (*n* = 17 for each group) (Moya et al., 2012). All groups exhibited significant swelling of the outer layer within 1 day. For conditions without covalent crosslinks (LVG with and without UV exposure, meth-LVG without UV exposure) the size of the outer layer decreased significantly from 1 to 2 days. By day 4 the outer layer was no longer observed. In contrast, the outer layer of the covalently crosslinked outer layers (meth-LVG with UV exposure) did not vary significantly after the initial swelling for the entire duration of the study, from a maximum of 251 ± 22 μm on day 4 to 226 ± 33 μm on day 40. Covalent crosslinking of methacrylated LVG increased the stability of the outer later in culture (Figure 8).

### *In vivo* Stability Model

**Figure 9:**
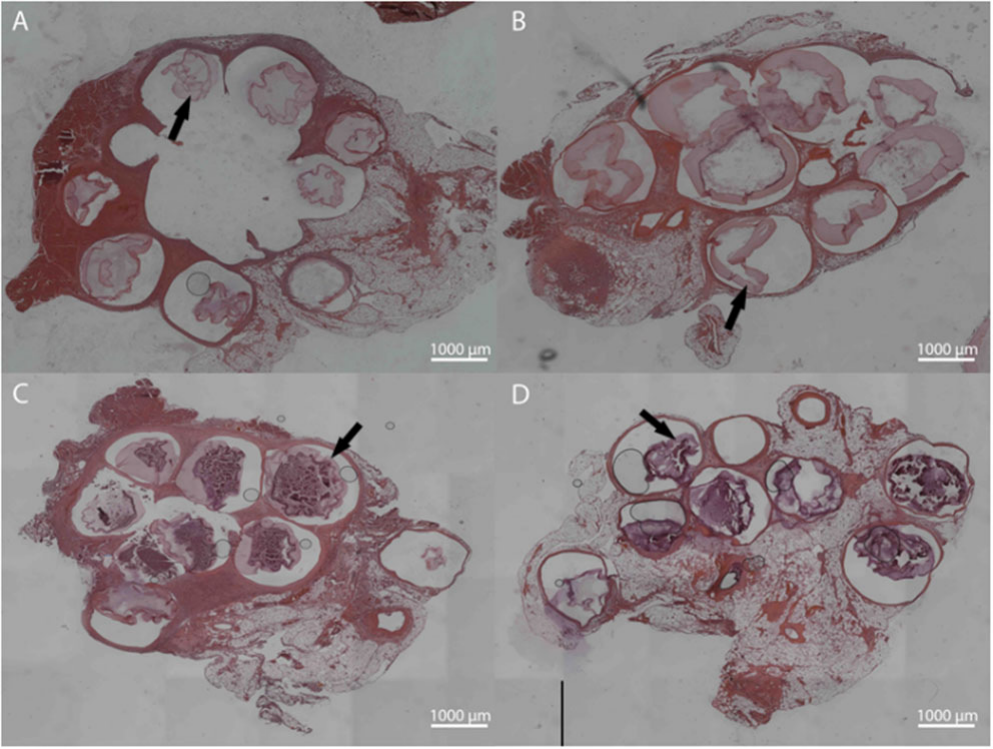
Hematoxylin and Eosin staining for LVG **(A,C)** and methacrylated LVG **(B,D)** at 1 week **(A,B)** and 3 weeks **(C,D)**. Intact microbeads observed for both groups at 1 and 3 weeks, with observable outer layer (arrows).

**Figure 10:**
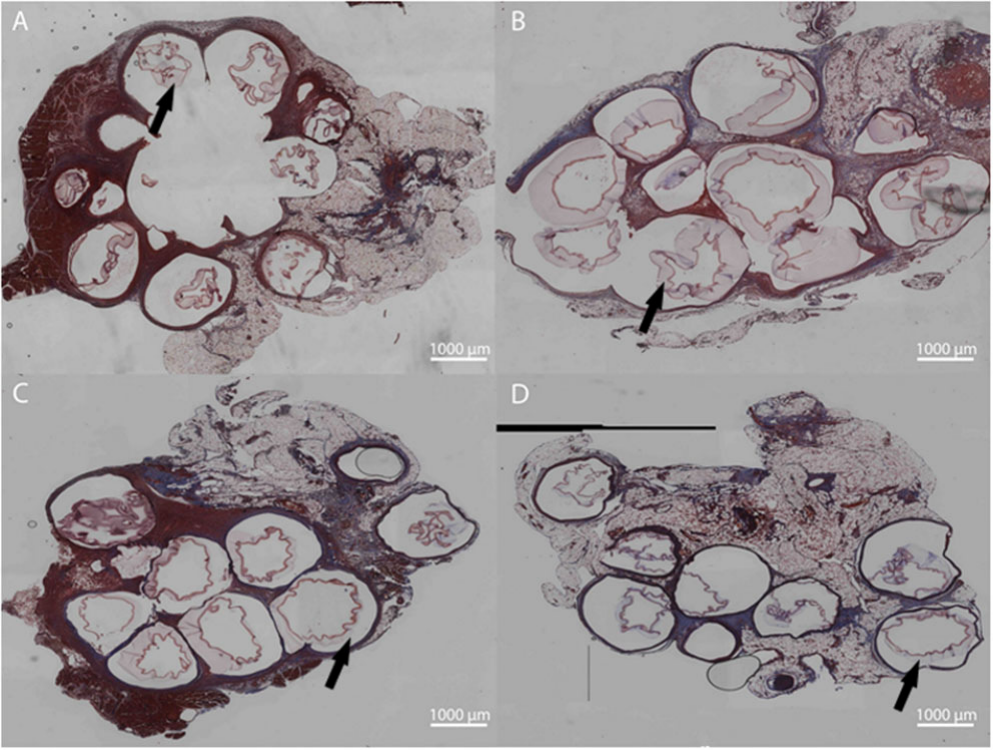
Masson's Trichrome staining for LVG **(A,C)** and methacrylated LVG **(B,D)** at 1 week **(A,B)** and 3 weeks **(C,D)**. Inflammation surrounding the microbeads are observed for both groups at all time points. Outer layers indicated with arrows.

**Figure 11:**
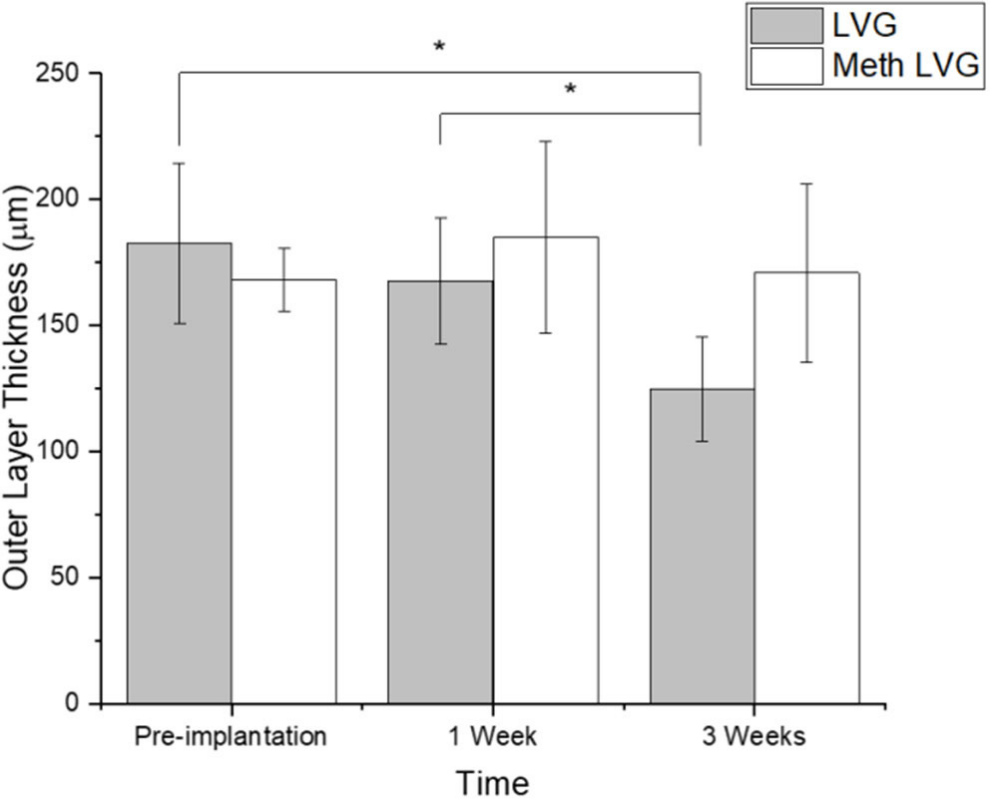
Outer layer thickness of alginate microbeads before (pre-implant) and after *in vivo* stability testing (1 and 3 weeks). *Denotes statistical significance (*p* < 0.05).

Microbeads formed with dual crosslinked (meth-LVG exposed to UV) and control microbeads (ionic crosslinks) were evaluated in a rat omental pouch model. During microbead implantation, 5 μg LPS was applied directly to the omentum to stimulate an inflammatory response in order to challenge the stability of the alginate microbeads. This concentration was selected from previous studies because it results in a robust inflammatory response that have been shown to break down alginate-based materials without systemic toxicity (Somo et al., 2018). At weeks 1 and 3, the microbeads were harvested and processed for histological analysis. Beads of each group were observed within the pouches at harvest. H&E and Masson's trichrome stains were performed on the samples harvested to evaluate microbead structure and inflammatory response. Alginate microbeads for both groups were observed within the tissue at both time points (Figures 9, 10). A robust inflammatory response was observed with multinucleated foreign body giant cells observed near the material surface. While histological processing can disrupt structure, the alginate microbeads were observed with the PLO layer, which appears as a thinner, dark purple line. The outer layer, which is the thicker, lighter pink section surrounding the PLO, was observed and quantified (Figure 11). The size of the outer layer at week 1 was 167 ± 25 μm and 185 ± 38 μm for LVG and methacrylated LVG, respectively. By week 3, the outer layer of the LVG beads had significantly decreased in size (*p* < 0.05) to 124 ± 20 μm, but the outer layer of methacrylated LVG beads was not statistically different, with a final size of 170 ± 35 μm.

## Discussion

The current standard treatment for diabetes is exogenous insulin, administered either manually or with a controlled pump device. Cellular therapies designed to reestablish glucose-sensitive insulin production are being developed as an alternative approach. Multilayered alginate microbeads have been investigated as a method of encapsulation to protect implanted allograft β-cell islets as a treatment for people with diabetes (de Vos et al., 2003b; Qi et al., 2008). Current literature suggests that the failure of these structures can be caused by a variety of issues, from a lack of oxygen delivery, variations in transplantation site, biocompatibility, or degradation of the encapsulation material. Several attempts have been made to increase the stability of the A-PLO-A microbeads to account for these various mechanisms of failure (Sawhney and Hubbell, 1992; Chandy et al., 1999; Eiselt et al., 1999; Lee et al., 2000; Mahou et al., 2012). This research focuses on the material properties of the alginate used for encapsulation or modulation of the local inflammatory response. Previous research has shown that dual crosslinking of alginate results in a more stable hydrogel even in the presence of a robust inflammatory response (Somo et al., 2018). However, the previous work focused on techniques that would enhance the stability of the inner alginate microbead. In this work methacrylated alginate was investigated as a way to produce a more stable outer layer.

In order to generate A-PLO-A microbeads with dual crosslinked outer layers, alginate was modified with AEMA to introduce methacrylate side chains. The methacrylate groups enabled the introduction of covalent crosslinks following UV exposure in the presence of an appropriate photoinitiator. The degree of methacrylation was kept to a minimum to allow native LVG alginate to form ionic crosslinks, which allows for an easy method for forming the outer layer. The covalent crosslinks are expected to enhance stability, as the ionic calcium crosslinks which give alginate its structure normally can be disrupted by the presence of sodium ions or other cations present in the *in vivo* environment. Improvement in outer layer stability may improve the overall performance of A-PLO-A microbeads, as breakdown of the outer alginate layer can result in exposure of the pro-inflammatory PLO or PLL polymer coating (Vandenbossche et al., 1993; Hall et al., 2011). Polymer rheology and biological properties of alginate-based materials depend on molecular weight. The alginate used in this study ranged from 75 to 200 kDa. With methacrylation efficiency at 2.16% the effect on molecular weight was assumed to be negligible. However, future studies will quantitatively evaluate polymer molecular weight distribution and rheology. The results of this study demonstrate the dual crosslinking of methacrylated alginate for outer layer A-PLO-A results in increased stability *in vitro* and *in vivo*.

Stability testing of methacrylated A-PLO-A microbeads showed that unlike A-PLO-A LVG microbeads with an only ionically crosslinked outer layer, the dual crosslinked alginate outer layer remained intact when exposed to calcium chelating agents. Three distinct layers are seen in SEM images of the methacrylated LVG group. The likely reason for the outer layer remaining, despite the chelation of calcium from the outer layer, are the covalent crosslinks. The chelating agent, sodium citrate, removes the calcium that initially allows for a hydrogel to form around the PLO. When the calcium is removed, the chains are soluble in aqueous solution. The methacrylated LVG contains additional covalent crosslinks that are not affected by the chelating agent. The methacrylated LVG alginate exposed to UV exhibited swelling comparable to other groups and remained in solution for up to 40 days.

The *in vivo* stability of alginate microbeads was tested based on a protocol described in previous work (Somo et al., 2018). LPS is applied directly onto the omentum at the time of implantation to test the stability of alginate systems through exposure to a sustained inflammatory challenge. This surgical method has been applied in the absence of LPS with the stability of the microbeads varying with the intensity of the inflammatory challenge (McQuilling et al., 2011; Khanna et al., 2013; Pareta et al., 2014; Appel et al., 2016; Ibarra et al., 2016; Brown et al., 2020; Shrestha et al., 2020) In the future it would be valuable to compare the stability of the microbeads upon exposure to a heightened inflammatory challenge, as observed in this study, to the native inflammatory response in the absence of LPS. The integrity of the LVG and methacrylated LVG outer layers were evaluated at 1 and 3 weeks. After 1 week, both groups had microbeads that remained intact with the microbeads visible in histological sections. The microbeads also remained intact through 3 weeks post-implantation. Inflammatory tissue surrounded the microbeads, including foreign body giant cells visible near the surface of the implants. Masson's trichrome staining also revealed a thin layer of collagen surrounding the microbeads. Quantitative analysis of the outer layer showed a decrease in outer layer size for the LVG group at 3 weeks as compared to preimplantation and 1 week. There was no statistical change in outer layer size for the methacrylated LVG group, showing that it was more stable than the non-methacrylated LVG group. These early time points may not be sufficient to comprehensively evaluate the improved stability but give insight into the potential of this method. A previous experiment conducted by our group compared single layered crude alginate microbeads to single layer methacrylated alginate beads using a similar LPS challenge. Non-methacrylated microbeads were implanted into the omentum site and had completely failed by 1 week. (Somo et al., 2018) Similar results were not observed in this study. Though the *in vitro* results showed improved stability with methacrylation of the outer layer because microbeads both with and without a methacrylated outer layer remained intact up through 3 weeks, the *in vivo* results are inconclusive. In addition, the analysis methods are limited due to the results of histological processing on bead structure.

While the reason that the non-methacrylated beads survived longer *in vivo* as compared to previous studies is not known, changes in the microbead size and type of alginate used to stabilize the outer layer may have been a factor. Previous literature has shown that, when implanted, larger sized microbeads >1,500 μm elicit an attenuated foreign body reaction response compared to smaller microbeads (Veiseh et al., 2015). This may have been a factor even with an LPS challenge for LVG outer layer microbeads. LPS induces an immediate inflammatory response that is present regardless of the material implanted. When the acute inflammation caused by the LPS does not endure as long, there is less persistent inflammatory tissue (neutrophils, macrophages, and pro inflammatory mediators) that can further break down the outer layer and possibly invade within the inner layer. Another possible factor is the purity and type of alginate used. The di-axial configuration of the G-groups of the LVG allow for a stronger interaction with the cation. The alginate used to coat the outer layer consisted of 60% guluronic acid units. As described by the egg-box model, when sufficient cations are present, the outer layer created using LVG alginate would have increased mechanical strength (Gombotz, 1998). The alginate used in previous studies consisted of crude alginate, where the number of guluronic and mannuronic groups are unknown and can have a wide variation. This crude alginate was more likely to break down when exposed to LPS.

Microbeads can become destabilized by mechanical stresses and inflammation, resulting in swelling and degradation. While the exact mechanism of failure is unknown, stabilizing the outer layer can potentially prevent failure of implants. In this study, the outer LVG layer of A-PLO-A microbeads was methacrylated for covalent crosslinking as well as ionically crosslinked with calcium, which created a stable barrier to inflammatory intrusion. The microbeads were tested *in vivo* using an LPS challenge omentum pouch model. Beads with a methacrylated LVG outer layer were more stable than beads without under an inflammatory challenge, showing no significance decrease in outer layer size. Covalent crosslinking of the outer layer may be an important addition to cell encapsulation protocols to enhance stability. However, further studies are needed to examine long term survival and function.

## Data Availability Statement

The raw data supporting the conclusions of this article will be made available by the authors, without undue reservation.

## Ethics Statement

The animal study was reviewed and approved by Institutional Animal Care and Use Committee of the Illinois Institute of Technology.

## Author Contributions

SS carried out the experiments, designed studies, interpreted data, wrote the paper, and edited the final document. EB designed studies, interpreted data, wrote the paper, and edited the final document. JB carried out additional experiments, performed data analysis, took the lead in writing the paper, and edited the final document. All authors contributed to the article and approved the submitted version.

## Conflict of Interest

The authors declare that the research was conducted in the absence of any commercial or financial relationships that could be construed as a potential conflict of interest.
